# Expression of NKp46 Splice Variants in Nasal Lavage Following Respiratory Viral Infection: Domain 1-Negative Isoforms Predominate and Manifest Higher Activity

**DOI:** 10.3389/fimmu.2017.00161

**Published:** 2017-02-15

**Authors:** Yonat Shemer-Avni, Kiran Kundu, Avishai Shemesh, Michael Brusilovsky, Rami Yossef, Mesfin Meshesha, Semaria Solomon-Alemayehu, Shai Levin, Orly Gershoni-Yahalom, Kerry S. Campbell, Angel Porgador

**Affiliations:** ^1^The Shraga Segal Department of Microbiology, Immunology and Genetics, Faculty of Health Sciences, Ben-Gurion University of the Negev, Beer Sheva, Israel; ^2^National Institute for Biotechnology in the Negev, Ben-Gurion University of the Negev, Beer Sheva, Israel; ^3^Institute for Cancer Research, Fox Chase Cancer Center, Philadelphia, PA, USA

**Keywords:** splice variants, natural killer, NCR, NKp46, respiratory viral infection

## Abstract

The natural killer (NK) cell activating receptor NKp46/NCR1 plays a critical role in elimination of virus-infected and tumor cells. The *NCR1* gene can be transcribed into five different splice variants, but the functional importance and physiological distribution of NKp46 isoforms are not yet fully understood. Here, we shed light on differential expression of NKp46 splice variants in viral respiratory tract infections and their functional difference at the cellular level. NKp46 was the most predominantly expressed natural cytotoxicity receptor in the nasal lavage of patients infected with four respiratory viruses: respiratory syncytia virus, adenovirus, human metapneumovirus, or influenza A. Expression of NKp30 was far lower and NKp44 was absent in all patients. Domain 1-negative NKp46 splice variants (i.e., NKp46 isoform d) were the predominantly expressed isoform in nasal lavage following viral infections. Using our unique anti-NKp46 mAb, D2-9A5, which recognizes the D2 extracellular domain, and a commercial anti-NKp46 mAb, 9E2, which recognizes D1 domain, allowed us to identify a small subset of NKp46 D1-negative splice variant-expressing cells within cultured human primary NK cells. This NKp46 D1-negative subset also showed higher degranulation efficiency in term of CD107a surface expression. NK-92 cell lines expressing NKp46 D1-negative and NKp46 D1-positive splice variants also showed functional differences when interacting with targets. A NKp46 D1-negative isoform-expressing NK-92 cell line showed enhanced degranulation activity. To our knowledge, we provide the first evidence showing the physiological distribution and functional importance of human NKp46 splice variants under pathological conditions.

## Introduction

Acute viral respiratory tract infections represent a major public health and economic problem, affecting all age groups and causing severe disease, especially in infancy and old age. Various respiratory viruses, such as respiratory syncytial virus (RSV), influenza viruses, parainfluenza viruses, and adenoviruses may cause respiratory illness. In particular, RSV is the leading cause of hospitalization for respiratory tract illnesses in young children (1). Cellular immune responses play a major role in viral clearance. However, they can play a pivotal role in contributing to the pathogenesis of chronic diseases, e.g., asthma and apnea, in some cases (2, 3). The reported clinical manifestations for viral respiratory infections are largely overlapping, suggesting common regulatory pathways. Despite extensive diagnostic investigations of the operative cellular immune responses to respiratory infections, the role of natural killer (NK) cells and their involvement in immune responses to acute upper and lower respiratory tract infections remain largely unknown (4). NK cells have been found to be the major (25%) immune cells in non-squamous nasal lavage (5), but the specific type of NK-cells or their activity were not studied.

Natural killer cell activity is regulated by a balance between signals delivered by inhibitory and activating receptors. The natural cytotoxicity receptors (NCRs) NKp46 (NCR1), NKp44 (NCR2), and NKp30 (NCR3), as well as NKG2D, are the main activating receptors involved in mediating NK cell function in health and disease (6, 7). NCR and NKG2D mRNAs are subjected to alternative mRNA splicing events, resulting in the generation of splice variants that could mediate different and even opposing functions for the same receptor. For NKp30, three splice variants were reported: NKp30a, NKp30b, and NKp30c. NKp30a and NKp30b are considered as immune stimulatory isoforms and lead to increased IFN-γ and TNF-α secretion, while NKp30c leads to IL-10 secretion and immunosuppression. NKp30 splice variants have been studied by us and others in cancer, viral infection, and pregnancy (8–13). Also, three splice variants for NKp44 were reported: NKp44-1, NKp44-2, and NKp44-3. We were the first to report that the ITIM-positive NKp44-1 splice variant could serve as an inhibitory receptor when co-incubated with target cells expressing PCNA (14), and our findings with regard to the NKp44-PCNA inhibitory axis were corroborated by others (15). In acute myeloid leukemia (AML), sole expression of NKp44-1 was associated with poor survival of newly diagnosed patients (14). A NKp44-1-dominant inhibitory profile predominated in healthy pregnancy gestation, but preponderance of a NKp44 activation phenotype, was associated with pregnancy disorder (10). For NKG2D, it was reported that a truncated human NKG2D splice isoform negatively regulates NKG2D-mediated function (16).

Interestingly, no studies of the function and distribution of NKp46 splice variants have been published to date. Here, we studied the expression of NCRs in nasal lavage following respiratory viral infection and observed that NKp46 was expressed abundantly. The NKp46 receptor has five main splice variants divided into a group that contains both extracellular domains (3 isoforms) and a group that does not express the D1 domain (2 isoforms) (11, 17). Interestingly, the D1-negative NKp46 splice variants were the predominant isoforms expressed in nasal lavage following viral infection. We further showed that the D1-negative NKp46 splice variants are more active in both a NK cell line and primary human NK cells.

## Materials and Methods

### Antibodies and Fusion-Ig Proteins

The following fluorochrome conjugated anti-human monoclonal antibodies were used for flow cytometry: anti-CD3 (PE, IQproducts), anti-CD56 (allophycocyanin, BioLegend), anti-CD16 (Pacific blue, BioLegend), anti-NKp46 (Alexa Fluor 647, clone 9E2, BioLegend), anti-NKp46 (iFluro 488, clone D2-9A5, Gen-Script), and anti-CD107a (BrilliantViolet 421, BioLegend). Purified anti-human monoclonal antibodies used for cell stimulation were anti-NKp30 (clone 210847, R&D Systems) and anti-NKp46 (clone D2-9A5). Monoclonal antibody D2-9A5 was generated using hybridoma technology described previously (18). HRP-conjugated goat anti-mouse IgG, Fcγ fragment-specific (Jackson ImmunoResearch Laboratories) antibody was used as secondary antibody. Recombinant NKp46 receptor Ig proteins, respectively, NKp46-D1 Ig, NKp46-D2 Ig, NKp46-Full Ig, and Human Fc were generated as described previously (19, 20).

### Generation of NKp46 Domain 1^+^ and NKp46 Domain 1^−^ isoform-expressing NK Cell Lines and Cell Culture

NKp46 full length, i.e., domain 1^+^ (isoform a; matching NM_004829.5) and NKp46-D2, i.e., domain 1^−^ (isoform d; matching NM_001242356.1) cDNA were N-terminal FLAG-tagged and cloned into pBMN-IRES-GFP retroviral vector. NK-92 cells (ATCC CRL-2407), derived from human NK cell leukemia were then transduced with the constructs to produce NK-92-NKp46-Full (NK92-46Full) and NK-92-NKp46-D2 (NK92-46D2) cell lines, respectively. Retroviral transduction was performed as previously described (21–24). NK-92 cell lines were then grown in Alpha-MEM medium (Gibco, Life Technologies) supplemented with 10% horse serum, 10% FBS, 0.2 mM myo-inositol (Sigma), 0.1 mM β-mercaptoethanol (Sigma), 0.02 mM folic acid (Fisher Scientific), 100 IU/mL of recombinant human IL-2 (PeproTech), and 1% penicillin/streptomycin (Life Technologies). HEK293T, SV40 large T Ag-transfected HEK293 cells (CRL-11268) were used as target cell lines. HEK293T cells were cultured in DMEM (Gibco, Life Technologies) medium supplemented with 10% FBS and 1% penicillin/streptomycin. Cell lines were grown in a 5% CO_2_ humidified 37°C incubator.

### Isolation and Culture of Primary Human NK Cells

Natural killer cell isolation was done from peripheral blood of healthy volunteer donors, who were recruited by informed consent as approved by the Ben-Gurion University of The Negev Institutional Review Board, using the RosetteSep Human NK cell Enrichment Cocktail kit (Stem Cell Technologies). After purification, cells were cultured in CellGro stem cell serum-free growth medium (CellGenix) supplemented with 10% heat-inactivated human plasma from healthy donors, 1 mM sodium pyruvate, 2 mM l-glutamine, 1× MEM non-essential amino acids, 1% penicillin/streptomycin, 10 mM HEPES (Life Technologies), and 300 IU/ml human IL-2 (PeproTech) (23, 25).

### Samples

Respiratory specimens, mainly nasal washes, were routinely obtained from hospitalized children with acute respiratory tract infection or from healthy children undergoing elective procedures (control group). Samples of nasopharyngeal washing (NPW) were tested for respiratory virus infections by real-time PCR (26). Briefly, nucleic acid extraction was performed using NucliSense EasyMag (Biomerieux, Marcy l’Etoile, France), according to the manufacturer’s instructions. One milliliter of aspirate was extracted into 50 µl of elution solution. Amplification was carried out in a final volume of 10 µl, using the RNA Ultrasense One-step qRT-PCR system (Invitrogen, Carlsbad, CA, USA) with 4 µl of nucleic acid and four sets of primers and probes to detect four viruses, and an internal control (IC) set (27). Samples positive for respiratory syncytia virus (RSV), adenovirus (ADV), human metapneumovirus (HMPV), or influenza A (FLUA H1N1 strain), along with healthy controls, were included in the study.

#### Ethics Statement

The number of the Ethics Committee approval is 0113-14-SOR. The approval is to get respiratory specimens from children under 5 years old (hospitalized or healthy controls undergoing elective procedures). It includes the requirement to sign a written informed consent by the parents.

### RNA Extraction, Reverse Transcription, Real-time Reverse Transcriptase Polymerase Chain Reaction (qRTPCR), and Primer Set Efficiencies

#### qRTPCR for NCRs

The sets of primers and probes used to detect the NCRs were all designed to span an intron or a splice junction, and thus test differentially spliced RNA transcripts, avoiding testing of DNA in the samples. The primers and probes were described in Table S1 in Supplementary Material. Each sample (extracted as described above, from NPW) was tested in parallel for NKp30, NKp44, and NKp46. Amplification was carried out in a final volume of 20 µl, using the RNA UltraSense One-Step qRT-PCR system (Invitrogen, Carlsbad, CA, USA) with 4 µl of nucleic acid and the relevant set of primers and probes. Samples that were positive for NKp46 were further analyzed for the NKp46 isoforms, specifically the presence of D1 domain, see Table S2 in Supplementary Material for primers–probes combinations.

### Flow Cytometry

Human primary NK cells (10^5^ cells/condition) were incubated with CD3-PE, CD56-APC, and CD16-Pacific blue mAbs (1 µg/ml) on ice for 1 h, washed and NK purity was checked. Primary NK cells from day 14, 21, and 28 culture were double stained on ice with anti-NKp46-Alexa Fluor 647 (clone 9E2) and anti-NKp46-iFluro 488 (clone D2-9A5) mAbs (1 µg/ml) for 1 h, washed and assessed for isoform development. Flow cytometry was done using on a Gallios instrument (Beckman Coulter) and FACS data were analyzed using FlowJo software (Tree Star, Inc.).

### Cell Stimulation and Flow Cytometry-Based Degranulation Assay

For the NK cell degranulation assay, 96 well U-bottom plates were coated with 1.5 and 5 µg/ml of anti-NKp46 mAb (clone D2-9A5) and anti-NKp30 (clone 210847) mAb overnight at 4°C. After washing, 10^5^ human primary NK cells were added for each condition along with BrilliantViolet 421-conjugated anti-CD107a mAb (1:400 final dilutions). After 4 h of incubation (37°C, 5% CO_2_), cells were washed and stained again with the same anti-CD107a mAb (1:400 final dilution) and anti-NKp46 conjugated with Alexa Fluor 647 (clone 9E2). Percentages of CD107a^+^ cells were assessed from both 9E2-positive and -negative population. The NK-92 cell line was transduced to express NKp46 full-IRES-GFP (NK92-46Full) and the NK-92 cell line expressing NKp46 D2-IRES-GFP (NK92-46D2) were separately co-incubated for 4 h (37°C, 5% CO_2_) with HEK293T cells using a E:T ratio of 1:1, 1:2, or 1:5 along with BrilliantViolet 421-conjugated anti-CD107a mAb (1:400 final dilutions). Then, the cells were washed and stained again with BrilliantViolet 421-conjugated anti-CD107a mAb (1:400 final dilutions). NK-92 cells expressing the same level of GFP (correlating with equivalent levels of NKp46 isoform expression) were gated and percentages of CD107a^+^ cells were analyzed. Flow cytometry was done using Gallios instrument (Beckman Coulter) and FACS data were analyzed using FlowJo software (Tree Star, Inc.).

### ELISA Assay

ELISA plates were coated overnight at 4°C with 1 µg/ml of recombinant NKp46 receptor Ig proteins (NKp46-D1 Ig, NKp46-D2 Ig, and NKp46-Full Ig) and Human Fc as control. After washing, the plates were blocked with skimmed milk followed by incubation with 1 µg/ml of anti-NKp46 mAb (clone 9E2 or clone D2-9A5). Detection was done using HRP-conjugated goat anti-mouse IgG (1:1000 final dilution). OD was measured at 650 nm with a Dynex Technologies MRX microplate reader.

### Statistical Analysis

Graphics and statistical analysis were performed using GraphPad/Prism5, OriginPro8, and Microsoft Office/Excel software. Statistical significance was calculated using Mann–Whitney *U* or Wilcoxon rank-sum test and One-way ANOVA. *p* < 0.05 was considered statistically significant.

## Results

### Expression of NCRs, NKp46 Isoforms, and Cytokines in Nasal Lavage

**Figure 1:**
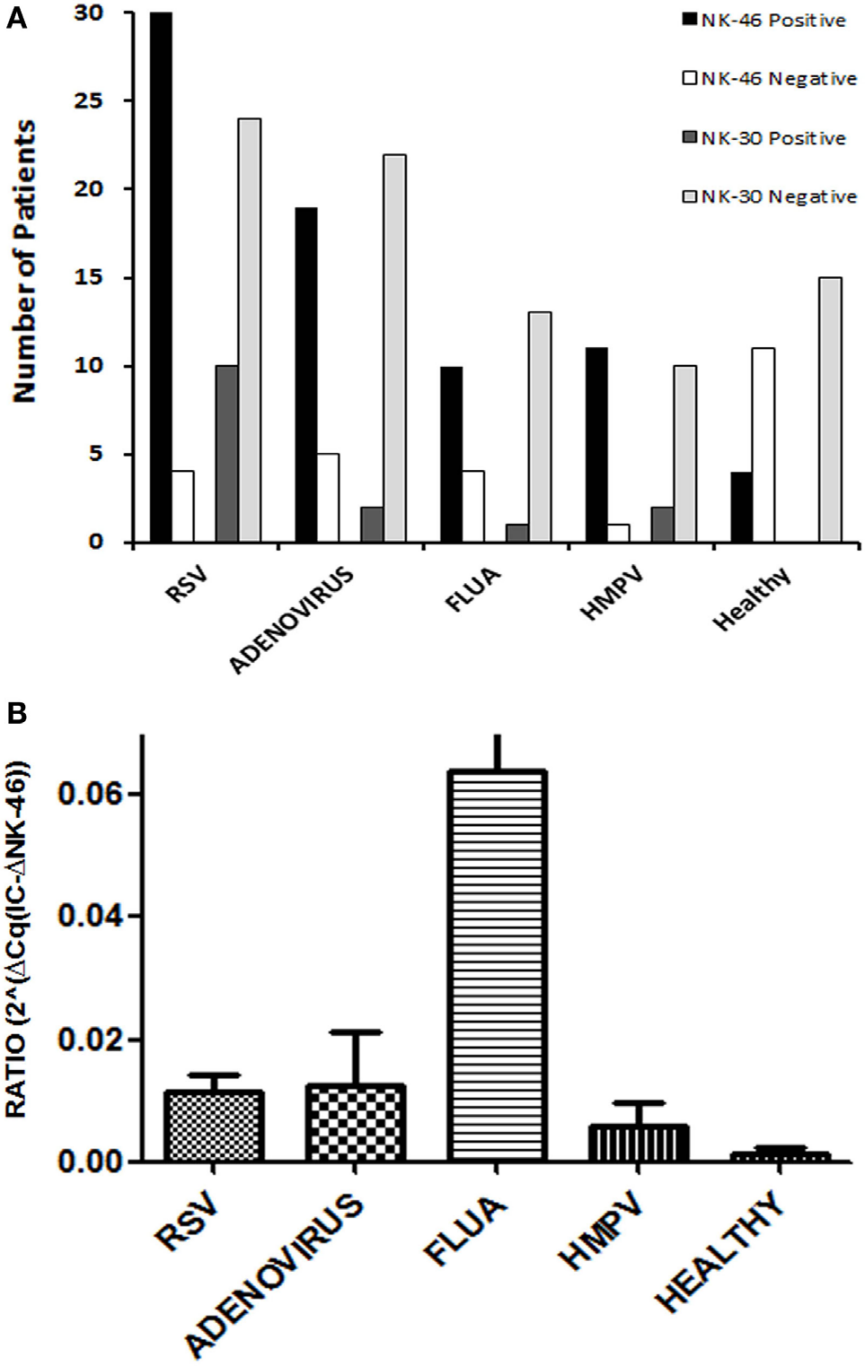
**Viral respiratory infections are accompanied by a specific pattern of natural cytotoxicity receptor (NCR) expression**. Nucleic acids were extracted from nasal wash samples, obtained from patients with respiratory viral infections, respectively, respiratory syncytial virus, adenovirus, human metapneumovirus, and influenza A, along with healthy controls and then were tested for mRNA expression of the three NCRs (NKp30, NKp44, and NKp46) by qRTPCR. **(A)** Depicted are the number of patients positive and negative for expression of NKp46, as well as NKp30. Purified PBMCs from healthy controls or whole blood served as a positive control in each test. NKp44 receptor expression was not found in any of the samples. **(B)** NKp46 mRNA expression was compared between healthy and each of the virus-infected groups. Graph bars show mean ± SEM. Statistical significance was calculated by Mann–Whitney *U* or Wilcoxon rank-sum test. *p* < 0.05 was considered as statically significant.

The assessment of NCR expression in the upper airways during viral infection was carried out using qRTPCR analysis. Three sets of primers and probe (Tables S1 and S2 in Supplementary Material) were designed for each of the three NCRs (NKp46, NKp44 and NKp30). Samples taken from nasal lavage (using nasal wash) of hospitalized patients with respiratory tract infection were tested. The most prevalent viruses that are found in these patients were chosen: 34 infected with RSV (respiratory syncytial virus) and 12 infected with HMPV, both ssRNA(+) viruses from the *Pneumoviridae* family; 24 infected with ADV, dsDNA viruses; and 14 infected with FLUA (influenza A) a segmented ssRNA(−) virus from the *Orthomyxoviridae*. Fifteen samples of healthy controls that arrived to the hospital for elective surgery were included in the study as well. Expression of the three NCRs was tested along with an IC, which is used to monitor for RNA extraction and for the PCR process (ERV-3 transcript, see Table S1 in Supplementary Material). To summarize, 83% (70 out of 84) of the infected samples were positive for NKp46 receptor, 18% (15 out of 84) were positive for NKp30, and none were positive for NKp44 (Figure 1A, detailing for the different viruses). All NKp30-positive samples were also positive for NKp46.

In the healthy control samples, 27% (4 out of 15) were positive for NKp46 and none was positive for NKp30 or NKp44 (Figure 1A). However, NKp46 expression in the nasal lavage of the infected samples was considerably higher as compared to the NKp46 expression in the nasal lavage of the four NKp46^+^ healthy controls (Figure 1B). The fact that NKp46 was widely detected in samples from infected individuals (88, 79, 71, and 92% for RSV, ADV, FLUA, and HMPV, respectively, Figure 1A), but not in most healthy individuals, suggests that NKp46 is a preferred receptor in the immune cells infiltrating the nasal lavage during viral infections regardless of the type of viral infection.

Natural killer cells, the prototypical member of group 1 innate lymphoid cells (ILCs) express NKp46 (28, 29) and can invade nasal lavage (30, 31).

**Figure 2:**
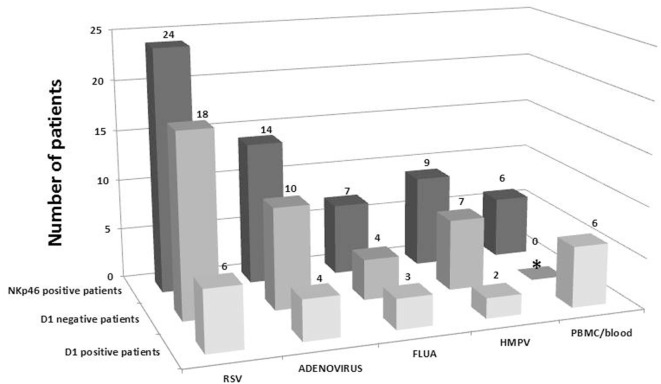
**Expression of D1-positive NKp46 isoforms in nasal wash is rare**. The presence or absence of NKp46 D1-positive isoforms in samples were tested using two sets of specific primers and probes that span exon4 and exon5 (for NKp46 total) and the D1-positive region (Exon3, see Tables S1 and S2 in Supplementary Material for details). Shown are the total number of NKp46 receptor-positive samples that were found in each type of virus tested, and distribution of D1-positive isoforms and D1-negative isoforms (calculated from the subtraction of D1-positive samples from the number of samples positive for total NKp46). Purified PBMCs from healthy controls or whole blood served as a positive control in each test. *Low levels of D1-negative splice variants were shown for PBMCs in a different qRTPCR assay with a Fw-primer targeting the conjugated Ex2–Ex4 sequence (following the exclusion of the Domain-1 coding Exon 3). However, in this Figure, D1-negative is calculated as described above.

We, next, investigated the expression of NKp46 isoforms in the nasal lavage following respiratory viral infection. Differential expression of NKp30 and NKp44 isoforms was reported for different diseases (8–10, 12–14, 16); yet, the distribution of NKp46 isoforms was not studied. The NKp46 receptor has five main splice variants divided into two groups; one group (3 splice variants) contains the domain 1 (D1) region (contains Exon 3) and the other group (2) does not contain the D1 region (lacks Exon 3) (17). We previously published that domain 2 (D2) but not domain 1 (D1), of NKp46 is involved in the recognition of cellular ligands and that recognition of viral hemagglutinins (HA) involve the hinge region connecting domain 2 to the NK cell membrane (32). Therefore, we assessed the expression of D1-positive NKp46 mRNAs in nasal lavage following respiratory viral infection. The samples were screened by qRTPCR, and the results are shown in Figure 2. In parallel, each assay included three positive controls: NK cell lines (positive control cell line that contains D1-negative and D1-positive NKp46 isoforms), isolated PBMCs, and whole blood samples of healthy adult individuals (the latter two are depicted in Figure 2). Figure 2 shows that most of the samples from the nasal lavage expressed NKp46 that lacked the D1 domain (75, 71, 57, and 77% for RSV, ADV, FLUA, and HMPV, respectively). In contrast, none of the positive controls was negative for expression of the NKp46 D1 isoforms (Figure 2 for PBMC/blood controls).

### NK-92 Cell Line Expressing the NKp46-Isoform d (D1-Negative) is Functionally More Active Than NK-92 Cell Line Expressing the Canonical NKp46-Isoform a (D1-Positive)

**Figure 3:**
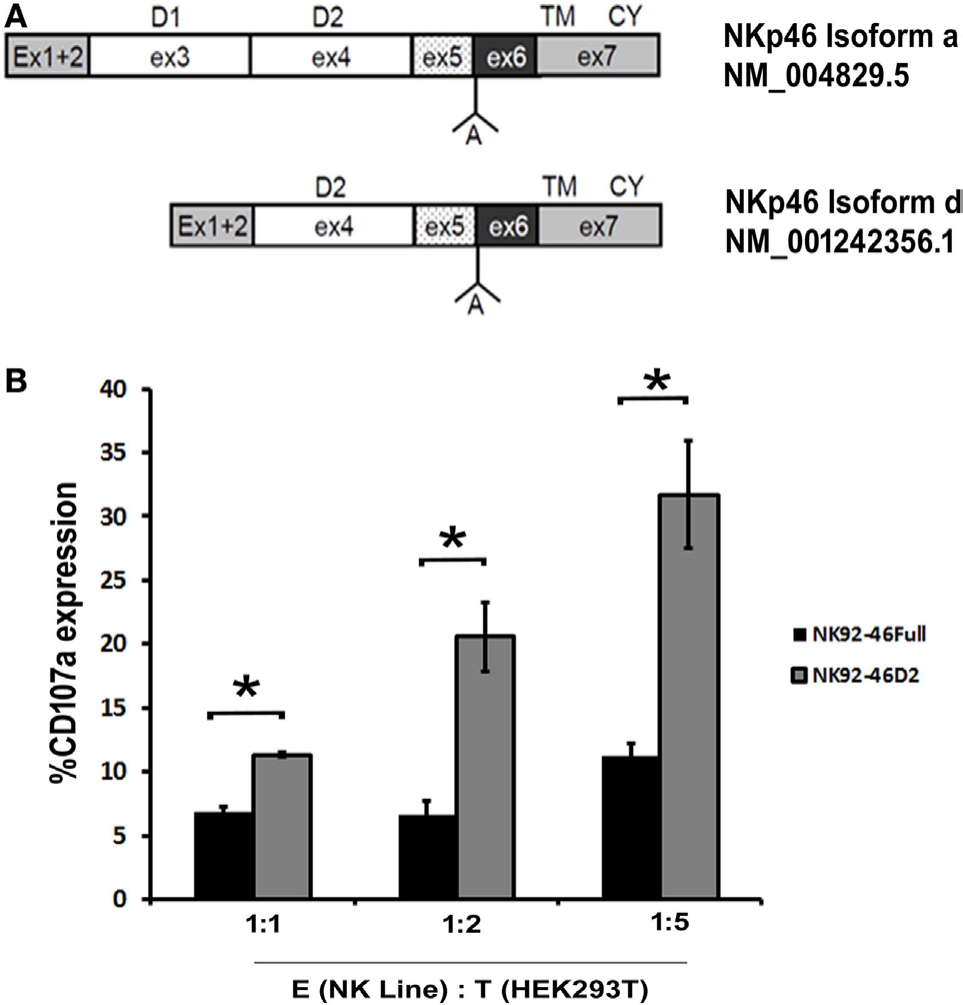
**Natural killer (NK)-92 cell line expressing the NKp46 D1-negative isoform is functionally more active than NK-92 cell line expressing the canonical NKp46**. **(A)** Schematic diagram of the canonical NKp46 receptor, i.e., NKp46 full (isoform a; matching NM_004829.5) and D1-negative splice variants of NKp46 receptor, i.e., NKp46 D2 (isoform d; matching NM_001242356.1). Domain 1(D1) within exon3, domain 2(D2) within exon4, transmembrane domain, and cytoplasmic domain (CY) within exon7 are marked. “A” shown between exons 5 and 6 note the Alanine AA existing in these isoforms. **(B)** To show degranulation efficiency, NK-92 cell line expressing NKp46 full, IRES-GFP (NK92-46Full), and NK-92 cell line expressing NKp46 D2, IRES-GFP (NK92-46D2) were co-incubated for 4 h with HEK293T cells then washed and stained for CD107a. Percentages of CD107a positive cells were analyzed by flow cytometry. The results are from three experiments. Graph bars show mean ± SEM. *p* < 0.05, ANOVA.

The NKp46 receptor consists of two Ig-C2-like ectodomains, D1 and D2 (33). As aforementioned, the NKp46 receptor has five main splice variants divided into a group that contains both ectodomains (3 isoforms) and a group that does not express the D1 domain (2 isoforms) (11, 17). We, thus, investigated the function of a representative from each group: the canonical NKp46 receptor, i.e., NKp46 full (isoform a; matching NM_004829.5) and the single domain NKp46-D2 (isoform d; matching NM_001242356.1) (Figure 3A).

We generated NK-92 cell lines expressing splice variants a (D1-positive) and d (D1-negative) using a lentiviral gene delivery approach (NK92-46Full and NK92-46D2, respectively) and studied degranulation activity of these two cell lines upon interaction with cancer cells. We measured the CD107a surface expression as a degranulation marker. NK92-46Full and NK92-46D2 were co-incubated with HEK293T cell line in an E:T ratio of 1:1, 1:2, 1:5 for 4 h and CD107a surface staining was performed. Since GFP was co-expressed with the transduced NKp46 splice variant, we could measure CD107a expression for NK92-46Full and NK92-46D2 expressing the same levels of transduced NKp46. We observed that for each E:T ratio, degranulation efficacy was significantly higher for NK92-46D2 as compared to NK92-46Full cells (Figure 3B, summary of 3 experiments).

### Cultured Human Primary NK Cells Develop a NKp46 D1-Negative-Dominant Subpopulation Manifesting Higher Activity

We previously developed the anti-NKp46 mAb, D2-9A5, which recognizes NKp46D2 (34). An ELISA assay to test for reactivity of the commercial anti-NKp46 mAb, clone 9E2, with recombinant NKp46D1-Ig, NKp46D2-Ig, and NKp46full-Ig revealed the recognition of NKp46D1 and NKp46full, but not NKp46D2 (Figure S1 in Supplementary Material). In contrast, D2-9A5 mAb recognized NKp46D2 and NKp46full, but not NKp46D1 (Figure S1 in Supplementary Material). Neither mAb reacted with negative control human Fc (Figure S1 in Supplementary Material). Similarly, the 9E2 mAb stained very positively NK92-46Full cells but to a significantly lesser extent the NK92-46D2 cells, while D2-9A5 mAb stained both cell lines similarly (Figure S2 in Supplementary Material). The low staining of NK92-46D2 by 9E2 antibody probably reflects the endogenous NKp46 expressed by NK-92 cells. Since both mAbs stain different domains, cross-reactivity is not a likely scenario. Yet, we did test for cross-reactivity by staining purified primary human NK cells either with each of the mAbs alone or by double staining with both mAbs. Figure S3 in Supplementary Material shows that the intensity of single staining with each of the mAbs was similar to its staining intensity in the double staining condition; thus demonstrating that 9E2 does not block D2-9A5 and *vice versa*. Therefore, we could use these antibodies together in flow cytometry analysis to test for the presence of a primary human NK subset in healthy controls that expresses only/mostly the D1-negative splice variant, as predicted from the RTPCR results from the nasal lavage of patients infected with respiratory viruses (Figure 2).

**Figure 4:**
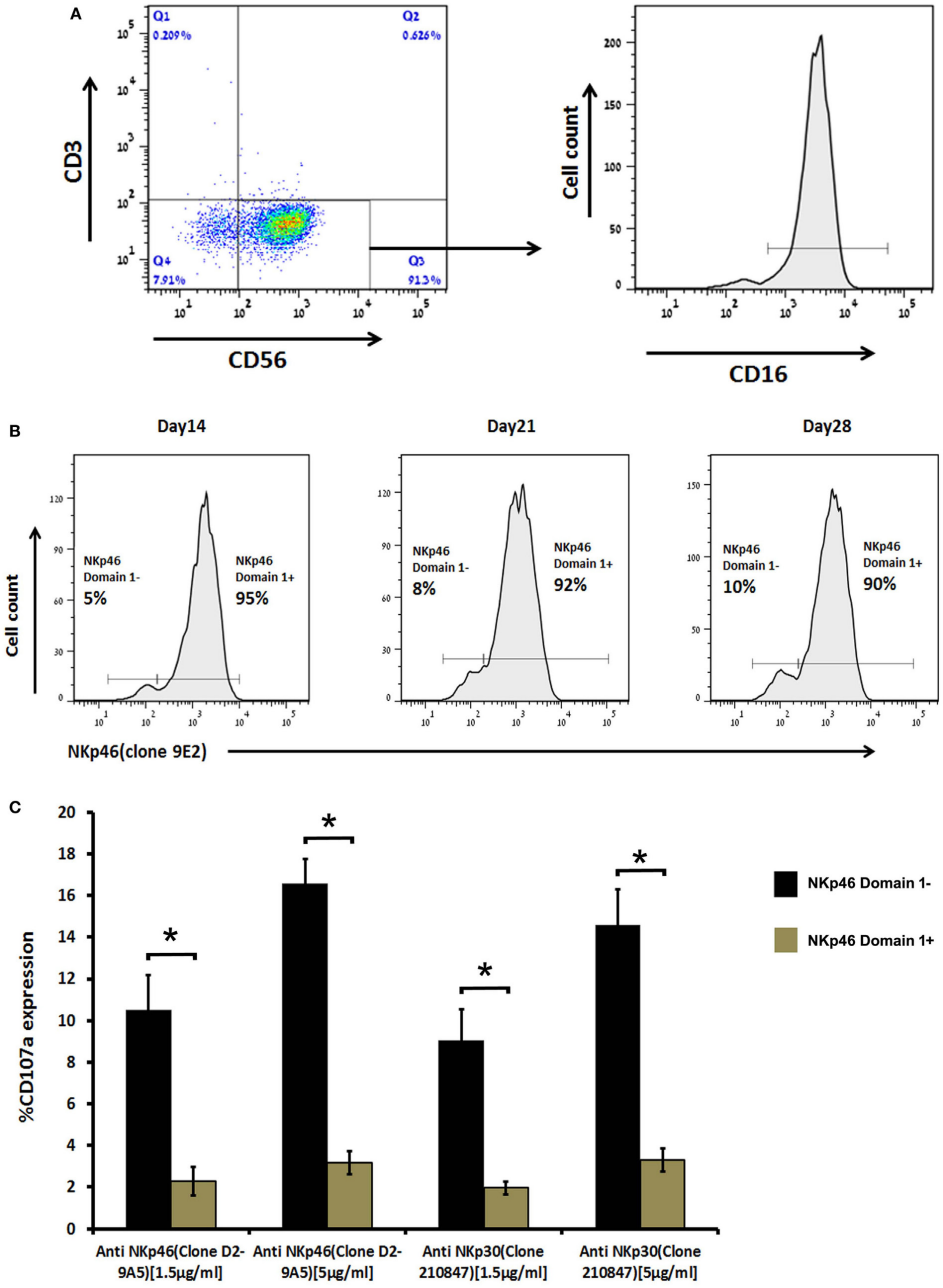
**Human primary natural killer (NK) cells when cultured *in vitro* became enriched with a distinct subpopulation expressing mostly the NKp46 D1-negative isoforms (NKp46 D2) that are functionally more active**. **(A)** Primary NK cells were stained with anti-CD3, anti-CD56, and anti-CD16 mAb to determine the percentage of CD3^-^ CD56^+^ NK cells and their CD16 expression. **(B)** Primary NK cells were double stained with anti-NKp46-Alexa Fluor 647 (clone 9E2) and anti-NKp46-iFluor 488 (clone D29A5) mAb at day 14, 21, and 28 of culture to show the development of NKp46 D1-negative isoforms. **(C)** When primary NK cells of day 28 culture were cross-linked for 4 h in a 96-well plate, coated with anti-NKp46 mAb (clone D29A5) and anti-NKp30 (clone 210847), then double stained with anti-CD107a and anti-NKp46 (clone 9E2), NK cells expressing the NKp46 D1-negative isoform became more active. Staining results were analyzed by flow cytometry. The results are from one representative experiment of triplicates. Graph bars show mean ± SEM (*p* < 0.05, ANOVA.

Since we could not retrieve live NK cells from nasal lavage of infected patients, we tested PBMCs. In freshly purified NK cells, we could not observe a subset that expresses only the NKp46D1-negative splice variants. Yet, when we cultured human primary NK cells in the presence of 300 IU of recombinant human IL-2, they gradually developed a small but very distinct subpopulation that express only NKp46D1-negative splice variants, i.e., cells that were stained with D2-9A5 mAb, but not with 9E2 mAb. The D2-9A5^+^9E2^−^ NK subset was evident at levels of 5% (14-day culture), 8% (21-day culture), and 10% (28-day culture) (Figures 4A,B). To investigate the functional relevance of this subset in primary NK cells, we assessed the degranulation potential of these cells as compared to the D2-9A5^+^9E2^+^ cultured NK cells. We stimulated the cells with anti-NKp30 or with anti-NKp46 (D2-9A5) mAbs and tested CD107a expression. Figure 4C shows that cultured NK cells lacking the D1 domain (D2-9A5^+^9E2^-^) were significantly more active as compared to cultured NK cells expressing the D1 domain (D2-9A5^+^9E2^+^). Taken together, our data reveal the evolution of NKp46 splice variants within primary NK cells over time, and their differential ability to release cytolytic granules.

## Discussion

We showed that NKp46 is not expressed regularly in the nasal lavage of healthy controls, but this receptor was highly expressed in the human nasal lavage from patients infected with various respiratory viruses (Figure 1). NKp46 is constitutively expressed by NK cells that are part of group 1 ILCs (28, 29). Other ILCs, such as intraepithelial (lung and intestine) ILC1 and NCR^+^ ILC3 subsets, also expressed NKp46 (28). Most non-NK ILC subsets that constitutively express NKp46, also manifest constitutive expression of NKp44; in contrast, peripheral blood NK cells express NKp44 only after stimulation. Therefore, our failure to observe any NKp44 expression in the human nasal lavage in parallel with NKp46 expression, indicates that the ILCs migrating to the nasal lavage upon respiratory nasal infection, are probably NK cells (35, 36).

Natural killer cells are capable of migrating to virus-infected tissue (30, 31) and specifically for IV infection, CCR2 was shown in mice to mediate the migration of NK cells during influenza virus infection (37). The immunopathology of upper respiratory tract viral infection is characterized by a complex interplay between respiratory virus, resident lung epithelial cells and infiltrating innate and adaptive immune cells. NK cells are one of the important immune cell types recruited into the infected respiratory tract to clear virus and resolve the infection. In addition to directly killing virus-infected cells, NK cells produce significant amounts of IFN-γ to augment cytotoxic T lymphocyte activity (31). NKp46 is a primary activating receptor mediating NK cell antiviral function (6, 7); a number of virus-derived ligands recognized directly by NKp46 have been reported. We have shown that NCRs, particularly NKp46, are involved in the functional recognition of viral HA and hemagglutinin-neuraminidases (38–41). Other groups have similarly reported the involvement of NKp46 and other NCRs in the recognition of viruses (42, 43).

The NKp46 receptor can be encoded by five major splice variants that can be divided into a group that contains both ectodomains (3 isoforms) and a group that does not express the D1 domain (2 isoforms) (11, 17). We showed that D1-positive splice variants are rare in the nasal lavage; therefore, the D1-negative splice variants are the dominant splice variants in the nasal lavage following respiratory viral infection (Figure 2). These results are in concordance with our previous reports that the NKp46 recognition site of viral ligands involve the membrane-proximal domain (domain 2) and the hinge peptide connecting NKp46-D2 to the cell membrane, but does not involve the membrane distal domain D1 (32). Differential tissue distribution of NCR splice variants has been previously reported for NKp30 and NKp44, but this is the first report of differential distribution of NKp46 splice variants. It was previously reported that NKp30 and NKp44 splice variant profiles differ between decidua basalis NK (dNK) cells and peripheral blood NK (pNK) cells. The NKp44-1 splice variant was shown to be significantly expressed in dNK cells compared to pNK cells, and the decidual cytokine milieu can change the splice variant profile of pNK cells to mimic that of dNK cells (11). Our recent observations also showed that a NKp44-1^dominant^ inhibitory profile predominates in dNK cells in healthy pregnancy gestation, whereas a NKp44-2/3^dominant^ activation profile proved to be more prevalent in cases of spontaneous abortions. No significant differences were observed in NKp30 splice variant expression profiles in dNK cells when comparing healthy gestation and spontaneous abortions (44). Recently we also reported that among the three different splice variants of NKp44, the inhibitory NKp44-1 isoform was significantly associated with poor survival of AML patients (14). In the case of gastrointestinal sarcoma tumors (GIST), distinct NKp30 splice variant profiles were observed in peripheral blood NK cells, as compared to healthy individuals (8). In that study, the immunosuppressive NKp30c isoform was expressed more prominently in individuals with GIST (8).

The differential distribution of NKp30 and NKp44 splice variants was associated with alterations in function of the different splice variants. In particular, inhibitory splice variants were identified and characterized for both NKp30 and NKp44 (8, 14, 44). In our studies with NK cell lines transfected with different NKp46 splice variants, we showed a functional difference between a D1-positive and a D1-negative splice variant, with a tendency of the D1-negative splice variant to exert a better NK cell degranulation response (Figure 3). Yet, this difference could not define the D1-positive splice variant as a suppressor variant. We were able to use anti-NKp46 mAbs that specifically stain domain 1 or domain 2, to identify primary human NK cells that more readily express D1-negative splice variants (Figures S1–S3 in Supplementary Material). NK cells expressing only the NKp46 D1-negative splice variants were negligible in peripheral blood; yet, following prolonged *in vitro* culture with IL-2, a small but distinct NK subset expressing mostly the D1-negative splice variants was observed. In accordance with the results from the NK cell lines, these D1-negative primary human NK cells exhibited a significantly better degranulation response, as compared to the other IL-2 cultured NK cells expressing both D1-negative and D1-positive NKp46 transcripts (Figure 4). We previously reported the evolution of a NK cell subset expressing a specific splice variant of NKp44 (14, 44), and the microenvironment of placenta, as well as tumor, was enriched with TGF-β and other cytokines that can actively modulate NKp44 splice variant expression. The development of NK cells expressing inhibitory NKp44 and NKp30 splice variants, orchestrated by cytokine milieu within placenta and tumor, provides a unique mechanism of immune tolerance.

The question whether or not the two splice variant groups (D1-negative and D1-positive NKp46 isoforms) are mutually exclusive, or can exist within the same NK cell, remained to be further explored. Yet, our D1–D2 antibody staining results of IL-2-maintained primary NK cells indicate the development of NK cell subset expressing mostly the D1-negative splice variants. Single-cell genomic analysis assays should be performed to assess the RNA splice variant distribution in the level of the single cell. Following the setting of single NK cell expressing mostly the D1-negative splice variants, the second subject to be resolved is whether the NK cells migrating to an active site are comprised mostly from cells expressing the same pattern of splice variants. Our results with the nasal lavage following respiratory viral infection point to a setting of NK cells manifesting similar phenotype of NKp46 D1-negative splice variants. Our recent studies with NKp44 splice variants indicate that dominant expression of splice variant (NKp44 isoform 1) can be observed in NK clones (14); moreover, we showed that NK cell subset in specific organs, e.g. the first trimester decidua in normal pregnancy, has the same splice variant profile which is mostly NKp44 isoform 1 (44).

To recapitulate, for the first time, this study demonstrates alterations in the distribution of NCR1 splice variants within nasal mucosa following respiratory viral infections, as well as the ability of cytokine stimulation to modulate the NKp46 splice variant expression profile, which may be representative of conditions in the infected respiratory tract. In addition, we also provide evidence for functional differences between the NCR1 splice variants, since the NKp46 domain 1-negative isoform proved to be functionally more active.

## Author Contributions

YS-A, KK, AS, and AP planned the experiments. MM, SS-A, AS, and SL performed the PCR experiments. KK, MB, and OG-Y performed the NK and FACS experiments. YS-A, KK, RY, AS, and AP analyzed the experiments. YS, KK, and AP wrote the manuscript. KC and AP edited the manuscript.

## Conflict of Interest Statement

The authors declare that the research was conducted in the absence of any commercial or financial relationships that could be construed as a potential conflict of interest.
