# Supplementary material for: Expression of NKp46 Splice Variants in Nasal Lavage Following Respiratory Viral Infection: Domain 1-Negative Isoforms Predominate and Manifest Higher Activity
Source: Front Immunol. 2017 Feb 15;8:161. doi: 10.3389/fimmu.2017.00161 (PMC5309248; doi:10.3389/fimmu.2017.00161)
Supplement: Supplementary file 1 [file Table_1.PDF]

Supplemental table 1: Primers and probes used for qRTPCR

Target	Primer/probe name	sequence
**NKp46**	NKp46-Ex4 Fw	5'-GGGACATACCGATGTTTTGG-3'
**NKp46 & D1-pos**	NKp46-Ex5 Rv	5'-AGGAAAGGTGGGGTCTTCAG-3'
**NKp46**	NKp46 Ex4-5 Probe (YAK)	YAK-5'CTGGTGTTCTCAATGTCGCCTGTGAC-3'-BHQ1
**D1-pos.**	NKp46-Ex3 Fw	5'-CCATCTGTTGCCAGGGAAAT-3'
**D1-pos.**	NKp46-Ex3 Probe (ROX)	ROX-5'-CAGCTGCACTTTGAAGGAAGCCTT-3'-BBQ
**NKp30**	NKp30 Fw	5’-TCACTGCTCAGATCCCCTTC-3’
	NKp30 Rv	5’-TCCAGGGTACGAATCTCAGG-3’
	NKp30 Probe	5’-/56-ROX/TTGATCATGGTCCATCCAGGATCCTG/3IAbRQSp/-3’
**NKp44**	NKp44 Fw	5’-TGATGCTGGCTTCTTCACTG-3’
	NKp44 Rv	5’-AGTCCAGGAGGTCTGTGTGG-3’
	NKp44 Probe	5’-/56-FAM/TCTGGTGGT/ZEN/ATCTCCAGCCTCTGCCT/3IABkFQ/-3’
**Human ERV-3**	IC-Fw	5’-CATGGGAAGCAAGGGAACTAATG-3’
	IC-Rv	5’-CCCAGCGAGCAATACAGAATTT-3’
	PHP (IC)	5’-CY5-TCTTCCCTCGAACCTGCACCATCAAT-BBQ-GTCA-3’

Supplemental table 2: qRTPCR sets up for NKp46 and D1-positive isoforms analysis

Target	Forward primer	Reverse primer	Probe
**NKp46**	NKp46-Ex4 Fw	NKp46-Ex5 Rv	NKp46-Ex4-5 Probe (YAK)
**D1-pos.**	NKp46-Ex3 Fw	NKp46-Ex5 Rv	NKp46-Ex3 Probe (ROX)
