# Supplementary figures and images for: Expression of NKp46 Splice Variants in Nasal Lavage Following Respiratory Viral Infection: Domain 1-Negative Isoforms Predominate and Manifest Higher Activity
Source: Front Immunol. 2017 Feb 15;8:161. doi: 10.3389/fimmu.2017.00161 (PMC5309248; doi:10.3389/fimmu.2017.00161)

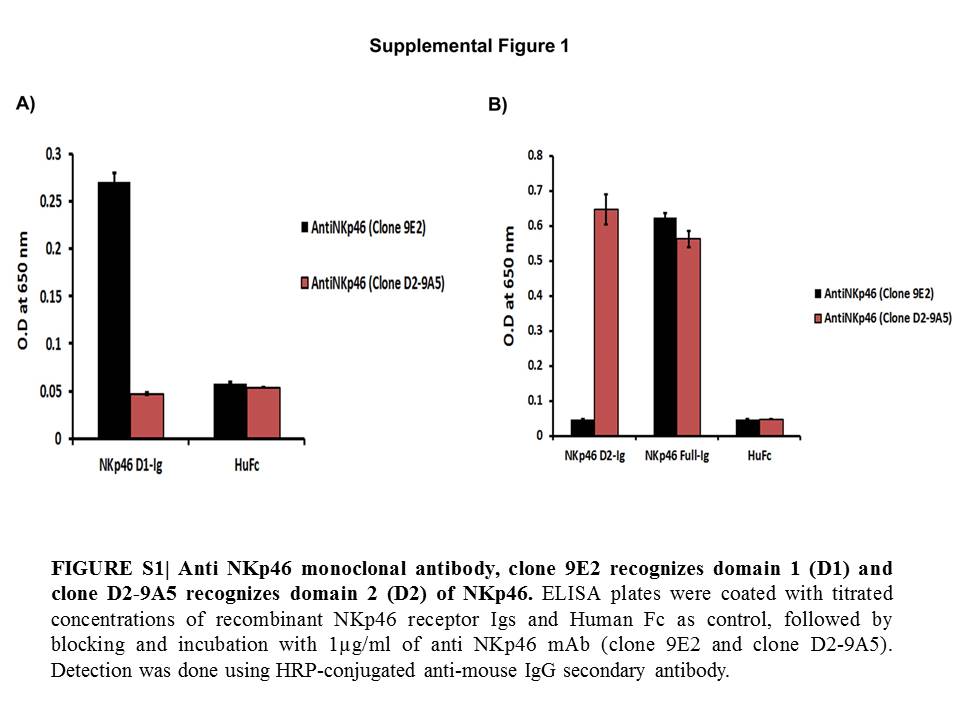

Supplement: Supplementary file 2 [file Image_1.JPEG]

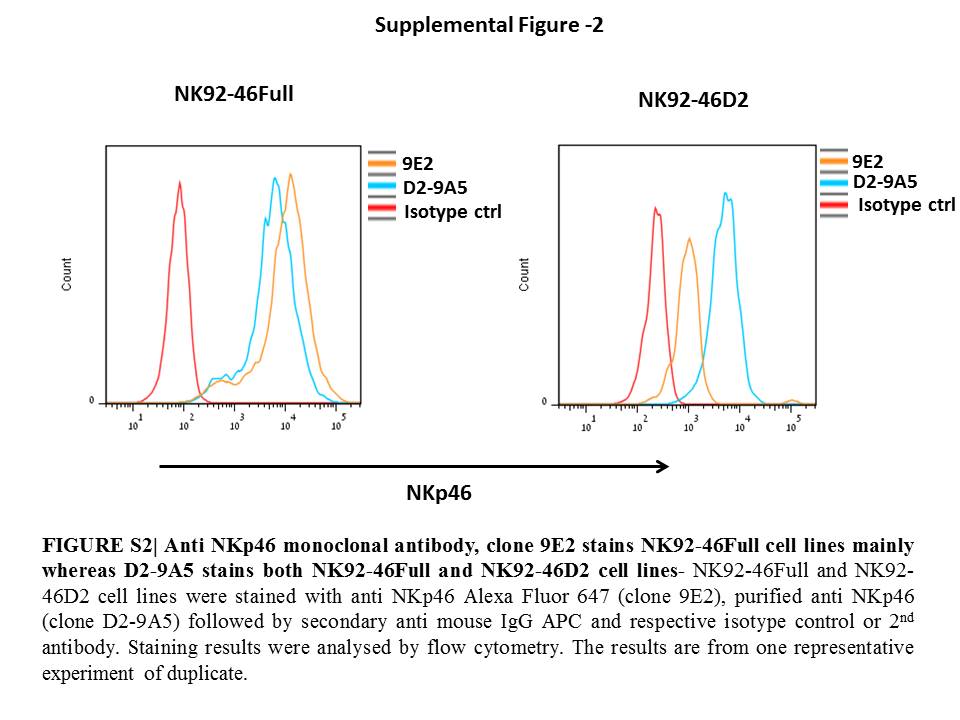

Supplement: Supplementary file 3 [file Image_2.JPG]

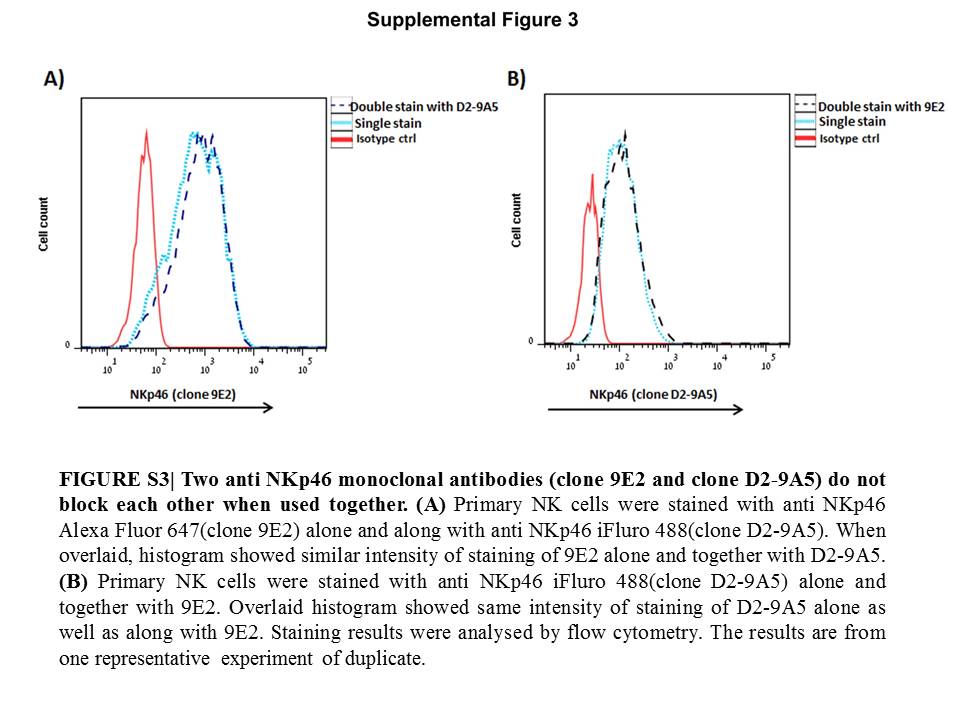

Supplement: Supplementary file 4 [file Image_3.JPEG]
